# Diversity and transmission competence in lymphatic filariasis vectors in West Africa, and the implications for accelerated elimination of *Anopheles*-transmitted filariasis

**DOI:** 10.1186/1756-3305-5-259

**Published:** 2012-11-14

**Authors:** Dziedzom K de Souza, Benjamin Koudou, Louise A Kelly-Hope, Michael D Wilson, Moses J Bockarie, Daniel A Boakye

**Affiliations:** 1 Parasitology Department, Noguchi Memorial Institute for Medical Research, University of Ghana, Legon-Accra, Ghana; 2 Centre for Neglected Tropical Diseases, Liverpool School of Tropical Medicine, University of Liverpool, Liverpool, UK

**Keywords:** Lymphatic Filariasis, Anopheles vectors, Vector-Parasite interactions, West Africa

## Abstract

Lymphatic Filariasis (LF) is targeted for elimination by the Global Programme for the Elimination of Lymphatic Filariasis (GPELF). The strategy adopted is based on the density dependent phenomenon of Facilitation, which hypothesizes that in an area where the vector species transmitting *Wuchereria bancrofti* are *Anopheles* mosquitoes, it is feasible to eliminate LF using Mass Drug Administration (MDA) because of the inability of *Anopheles* species to transmit low-density microfilaraemia. Even though earlier studies have shown *Anopheles species* can exhibit the process of Facilitation in West Africa, observations point towards the process of Limitation in certain areas, in which case vector control is recommended. Studies on *Anopheles* species in West Africa have also shown genetic differentiation, cryptic taxa and speciation, insecticide resistance and the existence of molecular and chromosomal forms, all of which could influence the vectorial capacity of the mosquitoes and ultimately the elimination goal. This paper outlines the uniqueness of LF vectors in West Africa and the challenges it poses to the 2020 elimination goal, based on the current MDA strategies.

## Review

### Introduction

The January 2012 London declaration on neglected tropical diseases (NTDs) [1] instilled renewed confidence in the global efforts to control or eliminate several NTDs, including lymphatic filariasis (LF). LF, is caused by the filarial parasites *Wuchereria bancrofti, Brugia malayi or Brugia timori* and is presently endemic in 72 countries [2]. Mosquito species belonging to the *Anopheles, Culex, Aedes, Mansonia, Coquillettidia* and *Ochlerotatus* genera are carriers of the LF parasites. In West Africa, *Anopheles* mosquitoes (vectors of malaria) are the main vectors of LF [3,4]. Although *Culex* mosquitoes have been suggested as vectors of LF [5,6], the data was insufficient to confirm that assertion. As such, there is minimal evidence that *Culex* mosquitoes contribute to the transmission of the disease. Current practices in the management of LF have been influenced by the push for integrated control of NTDs amenable to mass drug administration (MDA) [7] and the impact of vector control on LF transmission [8]. MDA coverage for LF increased from three million people treated in 12 countries in 2000, to more than 450 million in 53 countries in 2010 [9]. During that period, the disease was eliminated in China and Korea. Nine countries no longer require MDA [10] because of a natural decline in transmission intensity attributed to a range/ or multiple factors including vector control, provision of safe water, sanitation and hygiene. Vector control is now among the five strategies recommended by the WHO for prevention, control, elimination and eradication of NTDs in its 2012 road map for implementation [11]. Prior to 2012, the WHO strategy for LF elimination was based primarily on chemotherapy. However, the impact of vector control on LF transmission is becoming increasingly recognised [8,12-14]. Understanding the roles of different vectors in LF transmission and the implications for accelerated interruption of transmission in West Africa where the LF vectors are also targeted through malaria control efforts is important.

### LF control based on density dependent processes in the vectors

The Global Programme for the Elimination of Lymphatic Filariasis (GPELF) strategy is based on the mass drug administration (MDA) with Albendazole and either DEC or Ivermectin to reduce circulating microfilariae (mfs) below a threshold level, to break transmission by the disease vectors. The rationale supporting this strategy is based on results of research on vector-parasite systems that determine whether vectors will be effective in picking up and transmitting infection at low microfilaraemia levels [15]. These vector-parasite combinations are described under the density-dependent processes of “Facilitation” and “Limitation” [16]. Facilitation is the process where, below a certain threshold level of mfs, designated as Webber’s Critical Point [17], the transmission by anopheline vectors will be interrupted [18-20]. Limitation on the other hand represents a process whereby even at low mf levels there is stable transmission, which is found among culicines [4,21]. There is, however, a third case of non-regulated transmission by vectors, termed “Proportionality”. In this case, there is a constant percentage (linear relationship) of mf ingested by the vector during a blood meal developing to the infective stage. Limitation and Facilitation in vectors cause deviations from this linear relationship [21].

Limitation processes are linked with the fact that the number of parasites per mosquito cannot increase indefinitely. The relationship between mf intake and L3 output is linear at the onset and approaches a constant value or might decrease with increasing mf intake, as a result of excess mortality of vectors caused by ingestion of too many mf [21,22]. Thus in Limitation, there is a maximum threshold below which the limited process is ‘overefficient’ and above which it is ‘under-efficient’ [21]. This relationship has been observed in the culicines [4,18] which are believed not to be vectors of LF in West Africa, with the possible exception of *Mansonia sp*., recently reported to harbor infective mf in Ghana [23]. Thus in Limitation, the eradicability of LF is greatly impaired by shifting transmission thresholds towards lower values, requiring higher control efforts.

Facilitation processes on the other hand have been observed in anopheline mosquitoes [18,19], which are the vectors of LF in West Africa. Blood containing mf moves through the proboscis by a pumping action created by the cibarial and pharyngeal pumps. In some mosquito species, the pumps are lined with denticulate structures (cibarial armature) that can fatally damage passing mf [24]. At low mf densities, this cibarial armature substantially reduces the proportion of surviving mf. However, at high mf densities, the cibarial armature becomes inefficient because it is masked by a few mf promoting the survival of the others. Thus, at high mf densities transmission becomes efficient by shifting transmission thresholds towards higher values, which can more easily be achieved by control measures [21].

The assumption that it will be easy to use MDA alone in areas where *W. bancrofti* is transmitted by *Anopheles* species, including most endemic areas in Africa, was supported by results in Papua New Guinea (PNG), which showed that transmission by *An. punctulatus* was virtually eliminated after one year of treatment, even though the frequency of carriers in the human population ranged from 10.5% - 52.7% [25]. Also, one of the earliest documented cases of the elimination of LF occurred when indoor residual spraying with DDT to control malaria inadvertently eradicated LF in the Solomon Islands; here also the vector was an anopheline, *An. farauti*[17,21]. In contrast, in the Polynesian Islands of Moorea and Maupiti, where the vector was *Aedes polynesiensis,* over 50 years of MDA using DEC did not eliminate LF [17]. Despite these evidences and the assumption of vector-parasite phenomenon of Facilitation in *Anopheles*, there is current growing evidence that in certain areas, *Anopheles species* may be exhibiting the process of Limitation [26,27].

### Diversity of *An. gambiae* vectors of LF in West Africa

There is diversity among the vectors of LF, and therefore, it may not be practical to generalize based on data from PNG and the Solomon Islands. Threshold levels of microfilaraemia needed for the elimination of anopheline transmitted *W. bancrofti* LF might differ from species to species. The major *Anopheles* vectors of LF in West Africa are *An. gambiae* s.l, and the *An. funestus* group [26]. These species complexes are made up of distinct species, which are morphologically indistinguishable and may occur in sympatric situations. For example, in Ghana, several sympatric *Anopheles* species are vectors [26,28,29], and these are likely to differ in their vectorial role and in their capacity to transmit low-density microfilaraemia. Results of earlier studies in sub-Saharan Africa on the quantitative relations of transmission intensity and microfilarial reservoir have been found to vary among members of the *An. gambiae* complex and *An. funestus*[30-32]. These variations may include the proportion of mfs ingested by *Anopheles* mosquitoes which are damaged by their cibarial teeth, the percentage of mosquitoes ingesting mf and host mf density and the percentage of mosquitoes infected or mf density per mosquito and numbers of mf per ml of host blood. In their paper that examined the epidemiological significance of these processes, Southgate and Bryan reported the presence of Facilitation in *An. gambiae/W. bancrofti* from The Gambia, Burkina Faso and Tanzania [19]. In the same paper, although Facilitation was indicated for *An. arabiensis/W. bancrofti* when data from The Gambia and Tanzania were combined, the results from Tanzania alone did not indicate Facilitation. This difference in results was attributed to the low numbers of mosquitoes studied. Similarly, the observed non-facilitation for *An. melas*, *An. merus* and *An. funestus* was also attributed to low numbers of mosquitoes examined. However, it is possible that the non-Facilitation observed for these *Anopheles/W. bancrofti* combinations could have been due to variation in transmission efficiency inherent in the vectors and not necessarily to the low numbers of mosquitoes dissected. Webber commented on the possibility of eradicating anopheline-transmitted filariasis but did not discuss the information on *An. melas*, *An. merus* and *An. funestus* as vectors in Africa [20]. Recent studies by Amuzu and colleagues (2010), aimed at examining the cibarial armature of *An. gambiae* M and S molecular forms and *An. melas* in an area endemic for LF in Ghana, showed significantly less number of cibarial teeth in the *An. melas* compared to the M and S forms of the *An. gambiae* s.s [27]. As such, it is very clear from the above that anopheline LF vectors in West Africa may differ in their capacity to sustain low-level microfilaraemia. Furthermore, in areas of Ghana where MDA has not been able to eliminate transmission after more than 7 years of intervention, the main vector is *An. melas* (Boakye unpublished reports to WHO).

The diversity in *Anopheles* vectors of LF in West Africa is well documented from studies of malaria vectors in this region. Five chromosomal forms namely; “Forest”, “Bissau”, “Bamako”, “Mopti” and “Savannah” have been described [33-35]. The Mopti form of *An. gambiae* s.s*,* for example, is believed to be more associated with *W. bancrofti*[36], and is a relatively poor vector of malaria compared with other species such as the Savannah form [37,38]. There is further evidence suggesting that cryptic taxa may exist within *An. gambiae* s.s due to observed inversions in the micromorphology of the second chromosome for different populations [39] and thus selective effects due to the increase in certain inversion arrangements may result [33-35]. To add to these, incipient speciation has been reported among members of the *Anopheles species* in West Africa [40,41], raising further questions as to why these are only reported in West Africa and not elsewhere on the continent [42,43].

Two widespread molecular types, termed M and S forms [40,44] have also been described among the *Anopheles gambiae* s.s. Recent evidence also suggests the existence of two distinct chromosomal forms within the M form [45]. In Mali and Burkina Faso, the M form corresponds to the Mopti chromosomal form, whereas sympatric populations of Savannah and Bamako belong to the S molecular form. However, the correspondence between chromosomal and molecular forms does not hold true elsewhere in West Africa, especially where the Forest chromosomal forms exist [40,46].

Insecticide resistance has also been reported among the various vectors of LF on the African continent. The pyrethroid resistance mechanism of *kdr* mutation had been found distributed in the M and S forms of *An. gambiae* s.s., [47-49]. DDT and pyrethroid resistance have been widely observed in Africa, in *An. gambiae s.s.* and *An. arabiensis*, with multiple-resistance mechanisms observed in West Africa [50-53]. These resistance mechanisms may inadvertently influence the density dependent processes and the vector competence of various *Anopheles* species. Studies have suggested that highly elevated esterases involved in insecticide-resistance may inhibit development of mf in *Culex*[54], and similar effects could occur in insecticide resistant *Anopheles*[55,56].

The variability in diversity of *Anopheles* vectors of LF in West Africa may also be influenced by climate effects, such as temperature and rainfall, which indirectly may influence the transmission of LF [56,57]. In a study to assess the environmental factors affecting the distribution of *An. gambiae* s.s. in Ghana and the effects on disease distribution, de Souza and colleagues noted that temperature was the key factor affecting the distribution of the M and S forms of the *An. gambiae* s.s and that the M was significantly correlated with LF, and more prevalent in the high LF areas compared to low LF areas [58]. West Africa is the only region with the highest number of ecological zones (Mangrove, Coastal Savannah, Guinea Savannah, Sudan Savannah, Sahel Savannah, Semi-deciduous Forest and Evergreen Forest) in the world, the impact of these ecologies on vector diversity and disease transmission dynamics should not be overlooked. As such, the climate impacts on the biology of disease vectors will greatly affect their importance. Thus, an understanding of biodiversity and the importance of vector ecology crucial for the successful control of vector diseases [56,57].

### Eradicability of lymphatic filariasis in West Africa

A single strategy of MDA has been advocated for the elimination of LF in Africa, notwithstanding the diversity in the vectors of the disease. Some evidence [59,60] suggests the need for vector control as a supplement to MDA, in some areas, to achieve the elimination target of GPELF. For this, an appropriate vector control strategy to complement MDA in Africa will have to take a cue from previous malaria control efforts [61,62]. Historically, success in combating malaria has been attributed to mosquito control; yet, in recent times, this strategy has largely failed due to various reasons, including the development of insecticide resistance [63], economic Limitations and gaps in the basic biological knowledge of these vectors [64].

Vector control is an important component of the control of vector-borne diseases. Early efforts, before the era of DDT to control pests and disease vectors, took an ecological approach in the form of physical modification of the environment, chemical control and personal protection [65]. After the introduction of DDT and the synthetic insecticides, this approach lost its prominence and vector control became synonymous with insecticide use. The use of DDT and its analogs was heralded as the solution for the control of all insect pests, including vectors of human infection. This led to improper insecticide use, which had an enormous negative impact on non-target organisms causing a loss of biodiversity. Although most of the negative impacts resulted from insecticide application to control agricultural pests, vector control suffered as a consequence. This situation coupled with the development of vector resistance to most insecticides, and the high cost of new insecticides and their application led to a loss of interest in vector control and put emphasis on chemotherapy [66].

The GPELF is based on a strategy of MDA with Albendazole and DEC or Ivermectin, with the aim of reducing the parasite load in the human host, thereby preventing transmission, and a target of 80% coverage of the population at risk for at least 5 years [15] has been proposed. This ideal is not always achievable because of programmatic issues of drug distribution [67] and the perpetual threat of drug resistance [68,69]. Even if this level of coverage is achieved, the diversity of vectors and their differing abilities to transmit low level parasitaemia, may lead to a failure to stop transmission in some regions, after the interruption of MDA. In view of the above, vector control is now considered an important and integral part necessary to achieve elimination in specific areas where MDA alone will not provide the solution. This is especially important, with the very recent report of *Mansonia spp*. being very efficient vectors of LF, in Ghanaian communities [23], where they were previously thought to be non-vector species.

Furthermore, studies have suggested differing LF transmission efficiencies for the M and S molecular forms of the *An. gambiae* s.s [27,36], with the M form being a more efficient vector. Similar observations have been made with *An. melas* (Boakye *et al*., Unpublished), where *An. melas* is a more efficient vector than the *An. gambiae* s.s. Based on these findings we propose a model for the interruption of LF transmission in these different vector areas. Thus, areas with the predominant S form may require fewer MDA treatments. With the M form and *An. melas* exhibiting possible Limitation [24] areas with the predominant M form and *An. melas* may require longer treatment periods in addition to vector control measures. The implication of this, should it be tested and proven, will be in its economic importance. As such, in areas where there are high proportions of the *An. gambiae* S form, LF transmission may be interrupted using 3 to 5 rounds of MDA alone. On the other hand, LF transmission may require more than 5 rounds of MDA, and be complemented with vector control measures in areas with high numbers of *An. gambiae* M form and *An. melas*. Areas with equal proportions of M and S forms may also require additional vector control measures. This model, however, needs to be tested and evaluated in different vector areas. Countries like Guinea and Liberia that are yet to start MDA may provide the best settings for testing this model.

In the areas where vector control needs to be implemented, an integrated vector management (IVM) strategy targeted at the major vectors may needs to be adhered to and coordinated with MDA to give the best results at least cost [14]. LF fortunately shares the same vectors with malaria in most African countries and the practices for controlling the vectors of malarial parasites (the use of insecticide treated bednets, indoor-residual spraying) for personal and community protection - can at the same time be effective against both malaria and LF [70,71]. Even though the GPELF is based on MDA, vector control activities of the 'Roll Back Malaria’ campaign can considerably suppress the risk of *W. bancrofti* transmission in co-endemic areas.

## Conclusion

The use of current MDA alone campaigns, for LF elimination, in West Africa is based on two assumptions; 1. *Anopheles* species are the only vectors of LF in West Africa and 2. *Anopheles* vectors of *W. bancrofti* exhibit the vector-parasite process of Facilitation, based on which elimination is feasible through MDA alone. However, the recognition of different LF vectors in West Africa [23], with differing vector-parasite processes [26,27] and differing transmission efficiencies [27,36], all represent significant challenges to the GPELF 2020 objectives in the West African sub-region. Moreover, despite 5–8 rounds of MDA treatment, field reports have revealed persistent residual LF infections in some communities in Ghana and Burkina Faso [23]. Though reasons of non-compliance could be attributed to these residual infections [72], others have also hypothesized the influence by vector species [26,27]. It is also important to note that these observations may not be the same for every West African country, as factors such as ecology, species composition/diversity and insecticide resistance may influence vector transmission potential in different areas. Thus, an understanding of the vector competence of mosquitoes infected with *W. bancrofti* in different areas would be of particular interest and could be addressed using field or laboratory models. Nonetheless, despite these challenges, LF control efforts in West Africa should be supplemented with vector control, if the GPELF elimination goals of 2020 are to be achieved in West African countries.

## Competing interests

We declare that we have no conflicts of interest.

## Authors' contributions

DKD prepared the initial draft of the manuscript, and all other authors added their contributions and comments. All authors read and approved the final version of the MS.
